# Enrichment of beneficial bacteria in the skin microbiota of bats persisting with white-nose syndrome

**DOI:** 10.1186/s40168-017-0334-y

**Published:** 2017-09-05

**Authors:** Virginie Lemieux-Labonté, Anouk Simard, Craig K. R. Willis, François-Joseph Lapointe

**Affiliations:** 1 Département de Sciences Biologiques, Université de Montréal, CP 6182, Succursale Centre-ville, Montréal, Québec H2V 2S9 Canada; 2 Direction de l’expertise sur la faune terrestre, l’herpétofaune et l’avifaune, Ministère des Forêts, de la Faune et des Parcs, Québec, Canada; 3 Department of Biology and Centre for Forest Interdisciplinary Research, University of Winnipeg, Winnipeg, Manitoba Canada; 4 Quebec Centre for Biodiversity Science, CP 6182, Succursale Centre-ville, Montréal, Québec H2V 2S9 Canada

**Keywords:** Skin microbiota, White-nose syndrome, *Myotis lucifugus*, Endangered species, Conservation and management, 16S rRNA

## Abstract

**Background:**

Infectious diseases of wildlife are increasing worldwide with implications for conservation and human public health. The microbiota (i.e. microbial community living on or in a host) could influence wildlife disease resistance or tolerance. White-nose syndrome (WNS), caused by the fungus *Pseudogymnoascus destructans* (*Pd*), has killed millions of hibernating North American bats since 2007. We characterized the skin microbiota of naïve, pre-WNS little brown bats (*Myotis lucifugus*) from three WNS-negative hibernation sites and persisting, previously exposed bats from three WNS-positive sites to test the hypothesis that the skin microbiota of bats shifts following WNS invasion.

**Results:**

Using high-throughput 16S rRNA gene sequencing on 66 bats and 11 environmental samples, we found that hibernation site strongly influenced the composition and diversity of the skin microbiota. Bats from WNS-positive and WNS-negative sites differed in alpha and beta diversity, as well as in microbiota composition. Alpha diversity was reduced in persisting, WNS-positive bats, and the microbiota profile was enriched with particular taxa such *Janthinobacterium*, *Micrococcaceae*, *Pseudomonas*, *Ralstonia*, and *Rhodococcus*. Some of these taxa are recognized for their antifungal activity, and specific strains of *Rhodococcus* and *Pseudomonas* are known to inhibit *Pd* growth. Composition of the microbial community in the hibernaculum environment and the community on bat skin was superficially similar but differed in relative abundance of some bacterial taxa.

**Conclusions:**

Our results are consistent with the hypothesis that *Pd* invasion leads to a shift in the skin microbiota of surviving bats and suggest the possibility that the microbiota plays a protective role for bats facing WNS. The detection of what appears to be enrichment of beneficial bacteria in the skin microbiota of persisting bats is a promising discovery for species re-establishment. Our findings highlight not only the potential value of management actions that might encourage transmission, growth, and establishment of beneficial bacteria on bats, and within hibernacula, but also the potential risks of such management actions.

**Electronic supplementary material:**

The online version of this article (10.1186/s40168-017-0334-y) contains supplementary material, which is available to authorized users.

## Background

Infectious diseases of wildlife are on the rise worldwide with dramatic consequences for wildlife conservation and human public health [1–3]. In North America, insectivorous bats provide important ecosystem services by limiting insect pests and potentially saving billions of dollars annually for agriculture [4, 5]. However, a number of ecologically important species are threatened by white-nose syndrome (WNS). This skin disease, caused by the fungus *Pseudogymnoascus destructans* (*Pd*) [6, 7], has killed millions of North American bats since 2006 [8].

White-nose syndrome involves invasion of exposed skin by *Pd*, and the disease is defined by cup-shaped erosions and ulcerations on the tissue of the flight membranes (wings and tail), ears, and muzzle [9]. Infection of the flight membranes is thought to be the most pathologically significant aspect of the infection because this tissue is involved in fluid balance, thermoregulation, and gas exchange [10]. *Pd* invades hair follicles and sebaceous and apocrine glands [9]. This likely disrupts secretions that contribute to skin integrity [11, 12] with consequences for defense against pathogens and important skin commensal microorganisms [13]. Hibernating bats survive the winter on just a few grams of stored fat by using prolonged energy-saving bouts of torpor characterized by dramatically reduced body temperatures and metabolism [14–16]. *Pd* is adapted for growth at the low temperature characteristic of bat skin during torpor [6], and infection causes hibernating bats to warm up too frequently during winter and deplete their fat reserves [17, 18]. The immune system is downregulated during hibernation [19–21] which, in turn, facilitates infection.

Seven species of bats have suffered impacts from WNS in North America [22] but not all bat species are equally affected [23, 24]. It has been suggested that environmental conditions inside hibernacula, physiology, and behavior could all play a role in the variable tolerance of, or resistance to, infection with *Pd* among species [22, 24–26]. In Canada, the northern long-eared bat (*Myotis septentrionalis*), the little brown bat (*Myotis lucifugus*), and the tricolored bat (*Perimyotis subflavus*) are listed as federally endangered [22] due to mortality rates of 75–90% during the several-year invasion stage of the disease [27].

Despite extremely high mortality during the epidemic stage of WNS, some hibernating colonies of at least one highly vulnerable species (e.g., *M. lucifugus*) seem to have persisted following disease invasion [24, 28–30] with colony counts stabilizing at about 5 to 30% of their initial size [24, 28]. Recently, it was observed that intensity of infection with *Pd*, based on swabs of bat forearms and quantitative PCR, was significantly lower for persisting colonies in which *Pd* had become established, compared to colonies in the midst of the epidemic phase and massive declines [31]. One mechanism that could explain this pattern is a fundamental shift in the microbial community living on bat skin due to selection for *Pd* antagonists. Strong selection for microbial taxa that inhibit *Pd* could provide resistance to the fungus and increase bat survival.

Animal skin is an ecosystem inhabited by highly variable and complex communities of microorganisms [13]. This community, called microbiota, can be divided into a resident flora, defined as a relatively stable assemblage in size and composition, and a transient flora, acquired from the local environment and that only temporarily colonizes the skin [32]. A healthy skin microbiota can directly contribute to host fitness by occupying pathogen adhesion sites and producing pathogen inhibitors [13, 33]. Competitive interactions between beneficial and pathogenic skin microbes are hypothesized to play a role in disease dynamics for wild animals [34]. For example, the bacterium *Janthinobacterium lividum*, which lives on salamander skin, appears to confer resistance to the devastating fungal pathogen *Batrachochytrium dendrobatidis* [35] and could explain why some salamander populations decline while others do not. Recently, a strain of the bacterium *Pseudomonas fluorescens* isolated from the skin of a bat species thought to be resistant to WNS (*Eptesicus fuscus*) was shown to inhibit *Pd* growth in vitro [36] as well as in vivo for *M. lucifugus* [37]. It has been hypothesized that WNS could cause a shift in microbiota communities of the skin [38], and this could be one mechanism underlying resistance in persisting bats. However, it could also have negative consequences for bat populations if a shift in the microbiota makes it easier for opportunistic pathogens other than *Pd* to invade the skin. A detailed characterization of the skin microbiota for WNS-positive and WNS-negative bats is, therefore, needed to fully understand potential implications of skin microbial communities in the context of WNS.

Due to its direct exposure to the local environment, the skin microbiota is more dynamic and should be more strongly influenced by the environment, than the gut microbiota [39]. Environment and host species are strong predictors of variation in the skin microbiota among bats [40–42]. However, one study [43] found a strong influence of site and habitat type on the skin microbiota of 13 bat species in the western USA but was not able to detect an influence of host species or sex. For bats and amphibians, the local environment appears to act as a reservoir for skin microbiota, while conditions on the skin may lead to selection favoring or enriching particular taxa [40, 44, 45]. Consequently, host and local environmental factors appear to interact closely to shape the skin microbiota. This suggests that the skin microbiota could exhibit dramatic temporal variation for species characterized by seasonal shifts in physiology and habitat selection. Bats exhibit enormous changes in metabolism and habitat selection between winter and summer. Therefore, to fully characterize the skin microbiota and its relevance to WNS, bats must be sampled at the appropriate time during hibernation.

Several studies have reported on the skin microbiota of North American bats, but, to date, these have involved individuals not yet affected by WNS [42, 43] or have been based on relatively small sample sizes [38, 40]. Our objective was to understand the potential interaction between *Pd* and the skin microbiota of bats by comparing individuals from WNS-positive and WNS-negative regions. We used high-throughput 16S amplicon sequencing to characterize the composition and diversity of the skin microbiota of *M. lucifugus* sampled from WNS-positive (Québec) and WNS-negative (Manitoba) hibernacula in the northern part of this species’ range, in Canada. We tested two predictions of the hypothesis that WNS is causing selection favoring *Pd* antagonists on the skin microbiota of bats in the affected region. First, we predicted that bats persisting in WNS-affected sites would exhibit reduced diversity of their microbiota consistent with strong selection for a subset of pre-WNS microbial species [38]. Second, we predicted that the microbiota of persisting bats from WNS-affected sites would show a proportional increase in antifungal/anti-*Pd* bacterial species such as those identified in previous studies [36, 37, 46]. We also tested the third hypothesis that variation in the skin microbiota of hibernating bats relates to environmental variation in the microbial community of a given cave. We predicted that the diversity and composition of the microbial community living on individual bats would be similar to that found on substrates in the local environment of their hibernaculum and would differ from that on bats and the local environment in other hibernacula.

## Methods

### Sampling and ethics

During winter 2015–2016, we sampled the skin microbiota of 33 *M. lucifugus* from three WNS-negative hibernacula in central Manitoba (Canada) about 50 km north of the town of Grand Rapids (53° 30′ N; 99° 24′ W) and another 33 individuals from three sites known to be WNS-positive since 2010 in Québec (Canada) within 60 km north of Gatineau city (45° 28′ N; 75° 42′ W). The temperature within sites ranged from −3 to 7 °C at sampling time. Site and population information are specified in Table 1.

**Table 1:** *M. lucifugus* hibernaculum sites information in Manitoba and Québec provinces

Sites	Province	Hibernaculum	Rock	Pre-WNS count	Total count 2015–2016	Sampling dates
Abyss	Manitoba	Cave	Dolomite	NA	399	08/02/2016
Dale’s	Manitoba	Cave	Dolomite	NA	385	08/02/2016
Microwave	Manitoba	Cave	Dolomite	NA	30	09/02/2016
Emerald	Québec	Mine	Pyroxenite	735^a^	18	04/03/2016
Laflèche	Québec	Cave	Calcite	450^a^	155	23/11/2015
Lames	Québec	Cave	Calcite	Unknown^b^	105	24/11/2015

^a^2009–2010

^b^First count of 96 bats was in 2012–2013, after the arrival of WNS in the area

Bats in a given hibernaculum were always sampled from within the same area (i.e., room, gallery, corridor). We selected bats at random from among those we could reach from the ground. Little brown bats are highly gregarious during hibernation, and most individuals spend at least part of their time huddling or clustering during hibernation. We defined bats in direct contact with each other as being members of the same cluster. Sixty-four of the 66 bats we sampled were clustering with other bats, and cluster sizes ranged in size from 2 to 11 individuals. We sampled two bats at Emerald that were roosting solitarily (Additional file 1). We swabbed 11 individual bats per site. Samples were collected by swabbing in linear strokes the back and forearm of each bat for 20 s with a sterile Whatman Omniswab (Fisher Scientific) soaked in sterile 0.15 M NaCl [41]. Swab tips were ejected into MoBio Powersoil DNA isolation Kit tubes (MoBio Laboratories), which were transferred to −20 °C within 24 h of sampling until DNA extraction. Local environment samples were also collected by swabbing cave walls adjacent to clusters of sampled bats for 20 s in linear strokes (approx. 5 cm). As a negative control, a humidified sterile swab was exposed to open air for 20 s, prior to ejecting its tip into a MoBio tube.

Bats are vulnerable to disturbance during hibernation, and we were careful to minimize the impact of our visits. Only two people entered hibernacula for sampling, and bats were not handled during swabbing so we did not determine their sex. A previous study established that sex was not a significant predictor of the external microbiota of bats [43]. Therefore, differences among hibernacula are likely to reflect the influence of the local habitat (e.g., differences in temperature, humidity, and environmental bacteria), rather than difference in sex ratio among sites. All methods were approved by the Animal Welfare and Ethics Committee at Université de Montréal (Protocol Number #16-015) and the University of Winnipeg Animal Care Committee (Protocol Number AEO5639).

### DNA extraction, amplification, and sequencing

Bacterial genomic DNA was extracted from each swab using the MoBio Powersoil DNA isolation Kit according to the manufacturer’s protocol. Extractions were randomized for site and region to avoid detecting false patterns [47]. Extraction, amplification blanks, and the HM-782D Human Microbiome Project mock community (BEI Resources) were also included to detect possible contamination and assess sequencing accuracy [47, 48]. Amplification and sequencing were then performed as previously described [49]. Libraries were prepared using a two-step PCR. The first PCR amplified the hypervariable region V4 of the 16S small subunit ribosomal gene with forward primer U515_f: ACACGACGCTCTTCCGATCTYRYRGTGCCA GCMGCCGCGGTAA and reverse primer E786_R: CGGCATTCCTGCTGAACCGCTCTTCC GATCTGGACTACHVGGGTWTCTAAT [50]. Two microliters of extracted DNA (equivalent DNA amount by sample) was added to the PCR reaction containing 14.25 μl of sterile water, 5 μl HF buffer, 0.5 μl DNTPs, 0.25 μl Phusion High-Fidelity DNA Polymerase (New England Biolabs Inc.), and 1.5 μl of forward and reverse primers. Amplifications were performed with a Mastercycler Nexus GSX1 (Eppendorf) under the following conditions: initial denaturation at 98 °C for 30 s; 30 cycles alternating 98 °C for 25 s, 40 s at 54 °C, 35 s at 72 °C, and final elongation step for 1 min at 72 °C. Each sample was amplified in quadruplicate and pooled to limit possible PCR artifacts. All PCR products were then purified by PCR purification Agencourt AMPure XP (Beckman Coulter). The second PCR step consisted of adding primers containing a barcode (index) and Illumina adapter sequences to each DNA amplicon. To do so, 4 μl of the first step amplification product was added to a PCR reaction containing 10.25 μl of sterile water, 5 μl HF buffer, 0.5 μl DNTPs, 0.25 μl Phusion High-Fidelity DNA Polymerase, and 2.5 μl of forward primer PE-III-PCR-F:AATGATACGGCGACCACCGAGATCTACACTCTTT CCCTACACGACGCTCTTCCGATCT and reverse primer PE-III-PCR-001-096:CAAGCAGA AGACGGCATACGAGATNNNNNNNNNCGGTCTCGGCATTCCTGCTGAACCGCTCTTCCGATCT (N indicating the unique barcode) [51]. Indexing was performed under the following thermal conditions: initial denaturation at 98 °C for 30 s, 7 cycles alternating 98 °C for 30 s, 30 s at 83 °C, and finally 30 s at 72 °C. This second amplification was performed in triplicate. Samples were pooled and purified with the PCR purification Agencourt AMPure XP (Beckman Coulter). Qubit 2.0 Fluorometer (Invitrogen) was used to measure the DNA concentration of each sample. Indexed samples were then pooled to obtain a final concentration range between 10 and 20 ng/μl. DNA was next diluted and denatured according to the manufacturer’s protocol for paired-end sequencing using MiSeq Reagent Kit v2 (500 cycles) 2 × 250 bp on MiSeq (Illumina).

### Data analysis

We amplified 4,072,792 sequences classified into 13,224 operational taxonomic units (OTUs) from the 66 swabs of bat skin and 11 environmental samples (one or two per site). A total of 3,729,096 sequences classified in 11,812 OTUs were amplified from bat samples with a mean of 56,501 sequences per sample (range 9920–100,812). A total of 343,696 sequences classified in 9302 OTUs were obtained from the 11 environmental samples, with a mean of 31,245 sequences per sample (range 10,325–73,877). We were able to match all expected sequences in the mock positive control, except for *Helicobacter pylori*, which genus was nonetheless the most abundant in the compositional data (see Additional file 2). The genera or families of the 20 expected mock taxa were also the most abundant in the mock profile. We detected 36 false positives, with very low abundances (< 0.3%) (see Additional file 2). After filtering out OTUs with abundance values smaller than 3, sampling controls, extraction controls, and library negative controls were dominated by *Halomonas* (5–75%, mean of 56%) and *Shewanella* genera (1–26%, mean 18%) (see Additional file 3).

Preclustering, quality filtering, primer removal, merging of raw sequences, and postclustering dereplication were performed with the SmileTrain scripts [52] for 16S data processing using USEARCH v. 7.0.1090 [53]. Distribution-based clustering using the dbOTUcaller algorithm was performed to cluster sequences into OTUs by considering the distribution of DNA sequences across samples and distances between sequences [51]. The corresponding OTU table, providing abundances of bacterial taxa in the different samples was assigned with QIIME version 1.9.0 [54] using GreenGenes database release 13_5 [55]. For alpha diversity and compositional analysis of bat samples, mitochondrial and chloroplastic DNA sequences, as well as OTUs with abundance values smaller than 3, were filtered out, leaving 3,716,672 sequences classified into 9897 OTUs. In addition, the genera *Halomonas* and *Shewanella*, present in negative controls, were filtered out from all bat samples for compositional analysis, resulting in 3,145,399 sequences classified into 9575 OTUs.

The diversity of the skin microbial community (alpha diversity) of each sample was computed using the Shannon index [56]. The Shannon index, which includes both OTU richness and evenness, was selected due to its reduced sensitivity to sample depth differences [49, 57] (Additional file 4). R version 3.1.3 [58] was used for all statistical analyses. Log-transformed alpha diversity values were compared between WNS-positive and WNS-negative regions, using a linear mixed-effect model (*lme()* function), and significance was tested with *anova.lme()* of the *nlme* package [59]. Hibernaculum and clusters were included as a random effect. Variation in diversity among sites within the WNS-positive and WNS-negative regions was tested using a one-way ANOVA (function *aov()*) and post hoc Tukey test (function *TukeyHSD()*) of the package *stats* [58].

The change in diversity among skin microbial community (beta diversity) was calculated among skin microbiota samples and environmental samples. Two distinct phylogenetic distances, unweighted UniFrac (qualitative) and weighted UniFrac (quantitative) [60, 61], were computed on rarefied data, as such measures could be sensitive to differences in sequencing depth [62, 63]. UniFrac distances were computed from bat samples rarefied at 9886 sequences/sample and from environmental + bat samples rarefied at 9898 sequences/sample after retrieving OTUs in low abundance (<3 sequences). Computations were performed with the *phyloseq* package [64]. All beta diversity results were visualized with principal coordinates analysis (PCoA) [65] using the *ordinate()* function. The UniFrac distance matrix was checked with *is.euclid()* function of the *ade4* package [66] prior to the ordination to ensure that all distances were Euclidian and properly represented by PCoA [67]. When required, square-root transformations were applied to obtain distance matrices satisfying the Euclidian condition. All phylogeny-based UniFrac distances were calculated using a phylogenetic tree constructed with FastTree 2.1.8 [68].

To assess the influence of explanatory variables on the microbiota composition, we used distance-based redundancy analysis (db-RDA), a method intended to conduct a redundancy analysis (RDA) on distance matrices [69]. It is computed by first decomposing UniFrac distances (weighted or unweighted) into principal coordinates and then applying RDA to the corresponding principal coordinates using the *capscale()* function of the R package *vegan* [70]. Four distinct models were constructed to test the relative importance of (1) WNS status (i.e., WNS-negative vs. WNS-positive), (2) sampling sites (i.e., the six different hibernacula), (3) types of samples (i.e., bat samples vs. local environment samples), and (4) clusters (i.e., bat clusters within each hibernaculum). To better understand the relationships among explanatory models in the variation of the microbial assemblages, partial db-RDA was also computed [71]. This form of RDA allows for exploration of the contribution of an explanatory variable model while controlling for other explanatory models. Adjusted R-squared (*R* ^2^) values [72] were calculated to compare the explanatory power of such models containing different numbers of variables. Significance of db-RDA and partial db-RDA was tested via 9999 permutations with the *anova.cca()* function of the R package *vegan* ***.***

The microbiota composition was explored down to genus level to assess differences among hibernacula and between WNS-positive and WNS-negative sites. To emphasize these differences, Indicator Value tests (*IndVal*) [73] were performed on relative abundance data, using the 26 taxa with a relative abundance larger than 1% for the analysis. The *IndVal* indicator value is based on the comparison of occurrences and abundances of taxa across predefined groups of bats (e.g., grouped by sites or WNS status). The analysis for any given taxon is not influenced by other taxa present in the dataset. It provides an index ranging between 0 and 1, the maximum value indicating a taxon exclusively present in one group. *IndVal* is calculated as the product of *A* (specificity, i.e., the probability that a site belongs to the group given the fact that a given species is found in that site) and *B* (fidelity, i.e., the probability of finding a given taxon at a site when the site belongs to that group) [73]. The *multipatt()* function of the R package *indicspecies* [74] was used to compute indicator values, and significance was assessed with 9999 permutations of object between groups. The *p.adjust()* function of the R package *stats* was used to correct *p* values for multiple comparisons [75]. A corrected *p* value threshold of 0.05 was considered significant in all tests, and only significant taxa with a specificity of *A* ≥ 0.4 were retained as indicators.

## Results

### Alpha diversity in WNS-positive and WNS-negative regions

**Fig. 1:**
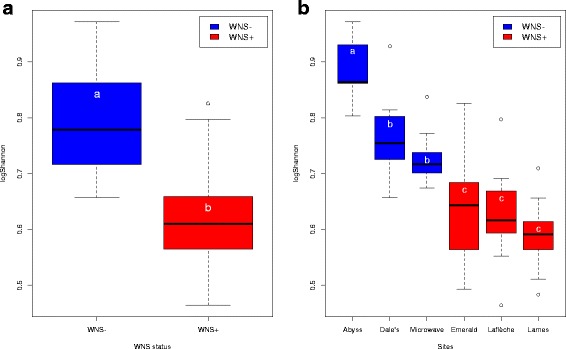
Alpha diversity of *M. lucifugus* skin microbiota in WNS-positive and WNS-negative sites in Canada. Distribution of alpha diversity within groups as estimated by the Shannon index for **a** hibernacula pooled by WNS status (positive vs. negative) and **b** all six hibernacula sampled in the study. Error bars represent standard deviations. Significant differences in alpha diversity among groups are indicated by different letters according to linear mixed model effect, ANOVA, and Tukey’s test (*p* ≤ 0.05)

After controlling for sites and bat clusters using a linear mixed-effects model, we found significant differences in Shannon diversity between our pooled set of WNS-positive hibernacula in Québec and WNS-negative hibernacula in Manitoba (*F*_1,4_ = 16.27, *p* ≤ 0.05) (Fig. 1a). WNS-positive sites had significantly lower Shannon diversity than WNS-negative sites (Fig. 1a). We also found significant variation in alpha diversity between some of the hibernacula in the WNS-negative region (ANOVA: *F*_2,30_ = 22.84, *p* ≤ 0.001), whereas all the three WNS-positive sites in Québec were statistically indistinguishable from each other and had relatively low alpha diversity (ANOVA: *F*_2,30_ = 0.96, *p* = 0.395) (Fig. 1b). Within the WNS-negative region in Manitoba, Abyss cave harbored particularly high alpha diversity and was significantly different from Dale’s (Tukey’s; *p* ≤ 0.001) and Microwave (Tukey’s: *p* ≤ 0.001).

### Beta diversity analysis of microbial community assemblage

**Fig. 2:**
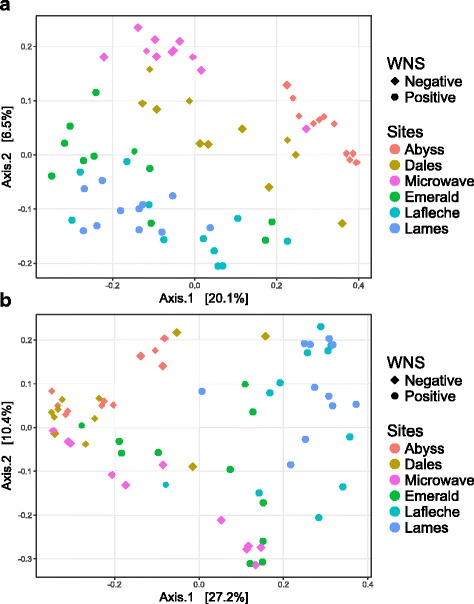
Principal coordinate analysis of *M. lucifugus* skin microbiota in WNS-positive and WNS-negative sites. **a** Principal coordinate analysis of unweighted UniFrac distances. **b** Principal coordinate analysis of weighted UniFrac distances. Each point represents a sample from an individual bat hibernating in one of the six different hibernacula that differed in WNS status

We first used beta diversity analysis to explore compositional differences among skin microbiota samples alone, that is, after removing all environmental samples from the analysis. The PCoA, based on unweighted UniFrac, revealed a clear separation between WNS-positive sites in Québec and WNS-negative sites in Manitoba, and also grouped samples from within the same hibernaculum (Fig. 2a). This pattern was not observed with weighted UniFrac (Fig. 2b), which implies that accounting for differential abundances (weighted UniFrac), and not just the presence/absence of bacterial OTUs between samples (unweighted UniFrac), affected our results. However, the first principal axes, accounting for 20.1% of the variation in the data, support the separation of microbiota samples according to WNS status (Fig. 2a).

In order to better relate these patterns to different variables, we used a distance-based redundancy analysis (db-RDA) to compute from UniFrac distances among skin microbiota samples using three distinct explanatory models: (1) WNS status, (2) sampling sites (hibernacula), and (3) clusters (bat clusters within each hibernaculum). Unweighted UniFrac distances revealed that each of these models explained a significant fraction of microbiota community variation (Table 2). The WNS status model explained 8%, sites explained 22%, and bat cluster explained 28% of microbiota community variation among samples. Weighted UniFrac distances accounting for abundance of taxa revealed similar patterns with WNS status explaining 14%, sites explaining 26%, and cluster explaining 30% of the variation in the microbiota samples (Table 2).

**Table 2:** db-RDA of unweighted and weighted UniFrac distances of *M. lucifugus* skin microbiota samples

	Model	Test	Adjusted *R* ^2^	*F* statistic
db-RDA unweighted UniFrac	WNS	Global test	0.0850***	7.0354
		Partial test: sites	0	0
		Partial test: clusters	0	0
	Sites	Global test	0.2204***	4.6754
		Partial test: clusters	0	0
		Partial test: WNS	0.1354***	3.7798
	Clusters	Global test	0.2840***	2.9829
		Partial test: sites	0.0637***	1.6676
		Partial test: WNS	0.1989***	2.4813
db-RDA weighted UniFrac	WNS	Global test	0.1413***	11.7000
		Partial test: sites	0	0
		Partial test: clusters	0	0
	Sites	Global test	0.2552***	5.4543
		Partial test: clusters	0	0
		Partial test: WNS	0.1138***	3.4457
	Clusters	Global test	0.3039***	3.1833
		Partial test: sites	0.0491***	1.5286
		Partial test: WNS	0.1623***	2.2433

WNS, sites, and clusters model redundant variation with UniFrac beta diversity variation among *M. lucifugus* skin microbiota. Global test for one model redundant variation on microbial community whereas the partial test for the model controlling variation from the other model. ***p ≤ 0.001. Total inertia of response variable matrix is 0.21286 for unweighted UniFrac db-RDA and 0.10555 for weighted UniFrac db-RDA

In light of these results, we conducted partial RDA to better distinguish the relative influence of our three explanatory models (Table 2). This analysis revealed that WNS status explained no variation after controlling for sites and/or clustering. Similarly, the sites model explained none of the variation after controlling for bat cluster. The cluster model, on the other hand, explained a significant fraction of variation in microbiota community after controlling for sites, both with weighted and unweighted UniFrac distances. These results suggest that bat clustering within each hibernaculum exerts strong influence on the composition of the skin microbiota. Controlling for WNS status, however, greatly reduced the variation explained by sites and cluster models alone, regardless of UniFrac distances (unweighted or weighted). Taken together, the results of simple and partial db-RDA analyses suggest that sites, combined with a local effect of bat clustering within sites, contribute to shaping the skin microbiota, whereas WNS status have much less influence.

**Fig. 3:**
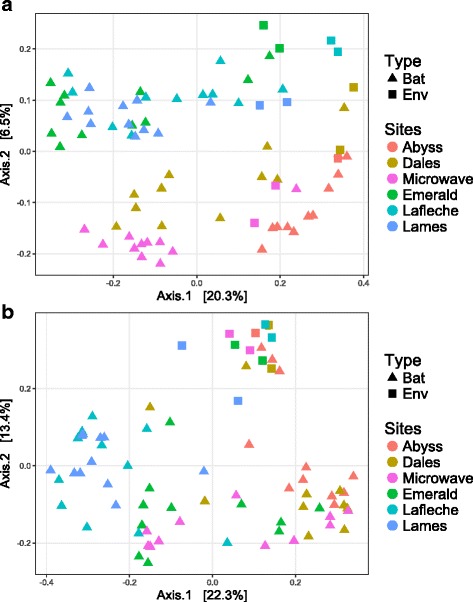
Principal coordinate analysis comparing local environment sites samples and *M. lucifugus* skin microbiota in Canada. **a** Principal coordinate analysis of unweighted UniFrac distances. **b** Principal coordinate analysis of weighted UniFrac distances. Each point represents a single sample

We next used unweighted and weighted UniFrac to explore the relationship between skin microbiota samples and environmental samples collected at each site. The first PCoA, based on unweighted UniFrac distances, grouped skin microbiota samples and environmental samples by sites, with some overlap between them (Fig. 3a). The second PCoA based on weighted UniFrac distances revealed a different pattern, however. In that case, the PCoA plot clearly distinguished between environmental samples and skin microbiota samples collected at all sites (Fig. 3b), except for three bat samples from Abyss and one from Dale’s. The results of PCoA suggest that the presence/absence of OTUs in bat skin samples is influenced by the local environment within each hibernaculum. Yet, the same analyses also reveal differences in abundance patterns of some OTUs in bat skin samples compared to local environmental samples.

We then used RDA to explore the influence of site (hibernaculum) and sample type (bat vs. environment samples) on variation in microbial community assemblage. Both of these models were significant (Table 3), but the sites model accounted for the most variation in the data, explaining 18% of the microbial community variation in both UniFrac distances employed (Table 3). The sample type model only explained 5% of microbial variation for unweighted UniFrac distances and 8% for weighted distances. The higher explanatory power of the weighted UniFrac model indicates that differences observed between environmental samples and bat samples partly depend on the relative abundance of each taxon within the corresponding microbial communities. This is consistent with patterns revealed by the PCoA plots (Fig. 3).

**Table 3:** db-RDA of unweighted and weighted UniFrac distances among local environment and bat skin microbiota samples

	Model	Test	Adjusted *R* ^2^	*F* statistic
db-RDA unweighted UniFrac	Sites	Global	0.1804***	4.3457
		Partial: type	0.1934***	4.8220
	Type	Global	0.0473***	4.7786
		Partial: sites	0.0604***	6.6491
db-RDA weighted UniFrac	Sites	Global	0.1763***	4.2544
		Partial: type	0.1892***	4.8584
	Type	Global	0.0752***	7.1866
		Partial: sites	0.0881***	9.5141

Sites and type model redundant variation with UniFrac beta diversity variation among *M. lucifugus* skin microbiota and site environmental microbial assemblage. Global test for one model redundant variation on microbial community whereas the partial test for the model controlling variation from the other model. ***p ≤ 0.001. Total inertia of response variable matrix is 0.21818 for db-RDA unweighted UniFrac and 0.22375 db-RDA weighted UniFrac

We used a partial db-RDA to better understand the relationship between the two explanatory models, and their influence on the composition of microbial communities. In both cases, when variation of one model was controlled for using partial db-RDA, the ability of the models to explain variation in microbial community composition was slightly increased by 1% (Table 3). These results suggest that both sample type and sites models had a non-redundant influence on microbial community variation and that the local environment is an important factor explaining skin microbiota patterns of hibernating bats in our study sites.

### Taxonomic indicators of WNS status

We found that the most abundant bacterial taxa were shared among all hibernacula, but we also identified indicator taxa present more often and more abundant at particular sites. At the phylum level, the dominant taxa accounting together for 86 to 98% of overall profiles in a given cave were Actinobacteria (23 to 53%), Proteobacteria (24 to 51%), and Bacteroidetes (6 to 38%) (Additionalfile 5). At the class level, the six principal taxa accounting for 80 to 98% of the total abundance were Actinobacteria, Gammaproteobacteria, Flavobacteriia, Sphingobacteriia, Alphaproteobacteria, and Betaproteobacteria (Additional file 6).

**Fig. 4:**
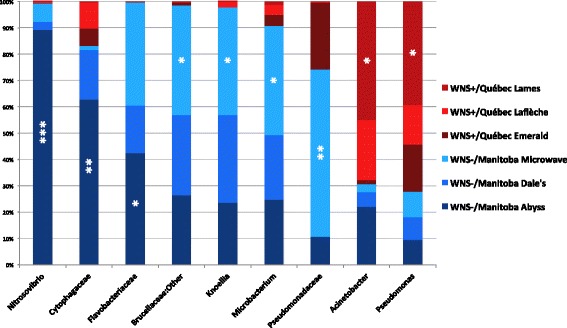
*M. lucifugus* skin microbiota taxa indicator of the six hibernacula of different WNS status in Canada. The sites from WNS-negative (Manitoba, Canada) and WNS-positive (Québec) regions are presented. The significant indicators were identified by *IndVal* analysis among the 26 more abundant taxa representing more than 1% of total abundance. Stars indicate hibernacula with significant representative taxa. *IndVal > 0.60, **IndVal L ≥ 0.75, ***IndVal ≥ 0.89

Generalist genera such as *Arthrobacter*, *Chryseobacterium*, *Flavobacterium*, Intrasporangiaceae, *Pedobacter*, *Mycoplana*, Pseudonocardiaceae, *Ralstonia*, *Rhodococcus*, Sinobacteraceae, and *Sphingobacterium* were identified from all sites (Additional file 7). Significant representatives were found among the 26 more abundant taxa representing more than 1% of the total composition profile (Fig. 4, Additional file 8). Among the more abundant taxa identified at three WNS-positive sites in Québec, only *Pseudomonas* and *Acinetobacter* were indicators of one site (Fig. 4, Additional file 8). On the other hand, the more abundant taxa at three WNS-negative sites in Manitoba, *Knoellia*, Brucellaceae:Other, *Microbacterium*, and Pseudomonadaceae were all indicators of the Microwave site (Fig. 4, Additional file 8). The largest indicator value was obtained for *Nitrosovibrio* at the Abyss site. Cytophagaceae and Flavobacteriaceae were also associated with Abyss. Representative taxa were identified from all hibernacula, except for Emerald, Laflèche and Dale’s.

**Fig. 5:**
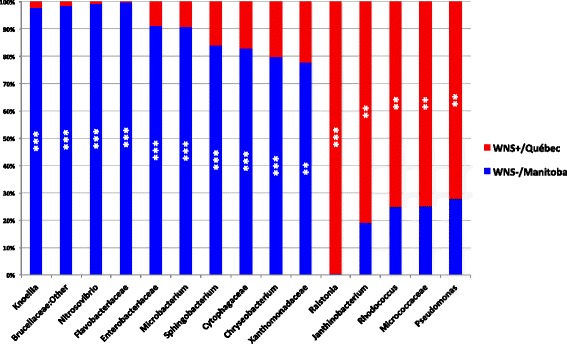
*M. lucifugus* skin microbiota taxa indicator of WNS-positive (Québec) and WNS-negative regions (Manitoba) in Canada. Significant indicators were found among the 26 more abundant taxa representing more than 1% of total abundance with *IndVal* analysis. Stars indicate regions with significant representative taxa. **IndVal ≥ 0.75, ***IndVal ≥ 0.89

We compared skin microbiota profiles based on WNS status in order to highlight possible differences in microbial composition related to the fungal disease. Here, again, some of the most abundant taxa such as *Pedobacter* and *Intrasporangiaceae* were not significant representatives as they were identified in both areas in similar relative abundance (Additional file 7). However, a large number of significant indicators were detected, some with high indicator values. At WNS-negative sites in Manitoba, significant indicators were *Knoellia*, Brucellaceae:Other, *Nitrosovibrio*, Flavobacteriaceae, Enterobacteriaceae, *Microbacterium*, *Sphingobacterium*, Cytophagaceae, *Chryseobacterium*, and Xanthomonadaceae (Fig. 5, Additional file 9). On the other hand, significant indicators of WNS-positive sites in Québec were *Ralstonia*, *Janthinobacterium*, *Rhodococcus*, Micrococcaceae, and *Pseudomonas* (Fig. 5, Additional file 9).

## Discussion

We compared the skin microbiota of bats from WNS-positive and WNS-negative sites to better understand the role of the microbiota as a factor in the host-pathogen interaction associated with WNS. We found support for the hypotheses that WNS has contributed changes in the skin microbiota for bats that are persisting in affected regions and that the skin microbiota is strongly influenced by the local environment within hibernacula. Although we cannot rule out the role of geographic variation confounded here with WNS status we believe that our results are more consistent with the proposal that WNS has led to a shift in the microbiota of bats inhabiting WNS-positive sites. For one, our analyses based on weighted UniFrac distances show no clustering environmental samples by province when analyzed together with bat samples, supporting that region is not a major driver of microbiota communities. Second, because *Pd* affects and interacts with the skin so directly and because higher levels of bacteria known to inhibit *Pd* growth in vitro [36, 46] and in vivo [37] were observed, it seems more likely that WNS status, and not geography, explains the compositional patterns. As predicted by our first hypothesis, the diversity of the skin microbiota was indeed smaller at WNS-positive sites compared to WNS-negative sites, which is consistent with a shift in microbiota caused by *Pd*. In addition, WNS status was a strong predictor of variation in Shannon diversity values across sites. A previous study on tricolored bats (*P. subflavus*) affected by WNS also revealed a trend for lower diversity values at WNS-positive sites [38], as shown here for little brown bats persisting after WNS invasion. Phylogenetic beta diversity analysis was also consistent with selection on the microbiota by *Pd* and WNS. Future studies, assessing diversity of the microbiota on bats from the same sites, before and after *Pd* invasion, would help resolve the WNS influence in microbiota diversity patterns of persisting bats.

At the compositional level, the skin microbiota of hibernating little brown bats is dominated by the classes Actinobacteria, Gammaproteobacteria, Flavobacteriia, Alphaproteobacteria, and Betaproteobacteria, a pattern consistent with previous investigation of the skin microbiota in several species of bats [42] and particularly *M. lucifugus* [40]. Our analysis also identified Sphingobacteriia as a predominant class. *IndVal* analysis revealed that one interesting genus, *Rhodococcus*, was significantly more abundant in skin microbiota samples collected at WNS-positive sites in Québec. This genus has previously been identified on bats [38, 76] and is known for its antifungal activity [77, 78]. Most interesting, a volatile organic chemical produced by *R*. *rhodochrous* strain DAP 96253 has been shown to inhibit *Pd* growth in vitro [46]. Several other genera, reported as antifungal agents, were also identified as significant indicators of bat samples collected at WNS-positive sites. Namely, *Pseudomonas* was enriched at all WNS-positive sites, whereas *Acinetobacter* was enriched at a single WNS-positive site. Both taxa are known for their antifungal activity [79, 80] and have been previously identified on the skin of North American bats [38, 40]. Moreover, one strain of *Pseudomonas fluorescens* has been shown to inhibit *Pd* growth in vitro and reduce disease severity and improve survival of bats with WNS in a laboratory challenge experiment [36, 37]. Another lesser-known antifungal bacterial genus, *Janthinobacterium*, was also identified as a significant representative at WNS-positive sites in Québec. Some species from the same genus isolated from the skin of wild amphibians confer resistance against the fungal pathogen *Batrachochytrium dendrobatidis* [35, 79]. Enrichment of multiple taxa with potential antifungal and anti-*Pd* activity in bats persisting following WNS invasion is consistent with our second hypothesis that the skin microbiota of bats provides a mechanism for resistance to, or tolerance of, *Pd* infection. Further studies should focus on any functional influence of these bacteria on the host-pathogen interaction between bats and *Pd*.

Consistent with our third hypothesis, we found that the skin microbiota of hibernating little brown bats is related to the microbial community composition of the nearby environmental substrates. That is, bacteria living on bats and bacteria living on adjacent cave walls are very likely exchanged by contact. However, bat skin samples and local environmental samples were by no means identical in their compositional profiles, indicating that microbial communities on the skin of hibernating bats are probably not regulated in the same way as in the environment (Additional file 10). These results are consistent with other studies of bats [40] or frogs [44, 45] showing that skin microbiota assemblages do not exactly mirror the microbial communities in the immediate environment. Although we did not detect any related variation within sites of microbial communities of the skin and that of the substrates, we found that individual bats strongly differ across sites. Moreover, the tendency for hibernating little brown bats to cluster, often in large groups of hundreds to thousands of individuals, is likely to reduce variation in the microbiota among individuals because of transfer within clusters, as shown by our analysis. Homogenization of the skin microbiota by close contact among individuals has also been observed in previous studies of bats and humans [41, 81]. Taken together, these results suggest that bat populations could differ in their susceptibility to WNS depending on the microbial community in their immediate environment, their reliance on clustering behavior, and the potential for clustering to homogenize the bacterial community. In this study, we did not attempt to quantify the potential influence of abiotic variables, such as pH, temperature, and humidity, and considered these factors as possible contributors to site effects. It would be interesting in future studies to analyze these factors separately to understand their relative influence on the microbial community on bats and in the environment. Temporal variation may also have influenced the compositional patterns observed in this study, but our experimental protocol was designed to reduce this effect as much as possible. All sites were sampled within a relatively short period of time (less than 3.5 months), and we avoided the start of hibernation when the experience of bats prior to hibernation might be expected to more strongly influence their skin microbiota. Moreover, the microbiota on bats or in the environment for the single WNS-positive site we sampled in March (Emerald) was not different from the two WNS-positive sites we sampled in November (Lames and Laflèche).

## Conclusions

This study highlights the role of skin microbiota for wildlife population health, conservation, and management in the face of emerging infectious diseases. The enrichment of potentially beneficial bacteria in skin microbiota samples collected at WNS-positive hibernacula is an encouraging discovery for the prospect of bat population recovery after WNS becomes endemic in a given region. This finding highlights the potential value of management actions that might encourage transmission, growth, and establishment of beneficial bacterial taxa on bats and within hibernacula [37]. However, our findings also highlight a potential risk of some proposed management actions. Considerable funding and time is currently being devoted to development and testing of potential chemical or biological treatments for WNS that could be applied to hibernating bats or hibernaculum substrates. Our results not only support previous work highlighting the potential of some bacteria as biological control agents for *Pd* (e.g., [36, 37, 46]) but also highlight a potential risk of biological or chemical treatments. Treatments that disrupt the skin microbiota or attenuate selection for a beneficial skin community could cause more harm than good for recovery of bat populations and the establishment of stable, long-term resistance to WNS in the wild. Thus, an important component of testing any potential treatment for WNS should be to confirm that it is selective for *Pd* and has minimal negative impacts on the whole non-target microbiota on bats or in hibernacula.

## Additional files

Additional file 1: Clusters of bats sampled within each hibernaculum and coded as dummy variables for db-RDA analysis.

Additional file 2: Positive control mock community analysis. Sequence set comparisons of the mock community to what is expected. File 1 shows the matching sequences and related taxa identified. File 2 shows the taxa composition of the mock in relative abundance, the matching taxa at the genus or family level, and the false positive taxa.

Additional file 3: Main taxa relative abundance (> 0.1%) in negative control samples. File 1 presents DNA extraction control samples, file 2 the library control, file 3 the negative site controls, and file 4 presents all controls together.

Additional file 4: Rarefaction curves of alpha diversity calculated on multiple rarefied data table for each of the 66 bat skin microbiota samples and 11 environmental samples. (A) Shannon diversity of bat skin samples. (B) Overall richness (OTUs observed) of bat skin samples. (C) Shannon diversity of environmental samples. (D) Overall richness (OTUs observed) of environmental samples.

Additional file 5: Main phyla identified in bat skin microbiota samples. The 8 more abundant phyla across all hibernacula are provided.

Additional file 6: Main classes identified in bat skin microbiota samples. The 6 more abundant classes across all hibernacula are provided.

Additional file 7: Major bacterial taxa identified in bat skin microbiota samples. The 16 more abundant taxa across all hibernacula are provided. Stars represent significant indicator taxa. *IndVal < 0.50, **IndVal ≥ 0.50, ***IndVal ≥ 0.89.

Additional file 8: *M. lucifugus* skin microbiota taxa indicator test and related association measure (*A*, *B*) of six hibernaculum groups with different WNS status in Canada. Indicator value tests were computed with the *multipatt*() function of the indicspecies package in R. Only taxa with *A* ≥ 0.4 were retained as indicators. *A*, the specificity, is the probability that a site belongs to the group given the fact that the species is found and *B*, the fidelity, is the probability of finding a given taxon when the sites belong to that group. *p ≤ 0.05, **p ≤ 0.01, ***p ≤ 0.001.

Additional file 9: *M. lucifugus* skin microbiota taxa indicator and related association measure (*A*, *B*) of WNS-positive (Québec) and WNS-negative (Manitoba) sites in Canada. Indicator value tests were computed with the *multipatt*() function of the indicspecies package in R. Only taxa with *A* ≥ 0.4 were retained as indicators. *A*, the specificity, is the probability that a site belongs to the group given the fact that the species is found and *B*, the fidelity, is the probability of finding a given taxon when the sites belong to that group. *p ≤ 0.05, **p ≤ 0.01, ***p ≤ 0.001.

Additional file 10: OTU table resulting from the analysis of 66 bat skin microbiota samples and 11 environmental samples.

## Abbreviations

*Pd*: *Pseudogymnoascus destructans*
WNS: White-nose syndrome
