# Supplementary figures and images for: Enrichment of beneficial bacteria in the skin microbiota of bats persisting with white-nose syndrome
Source: Microbiome. 2017 Sep 5;5:115. doi: 10.1186/s40168-017-0334-y (PMC5584028; doi:10.1186/s40168-017-0334-y)

**A**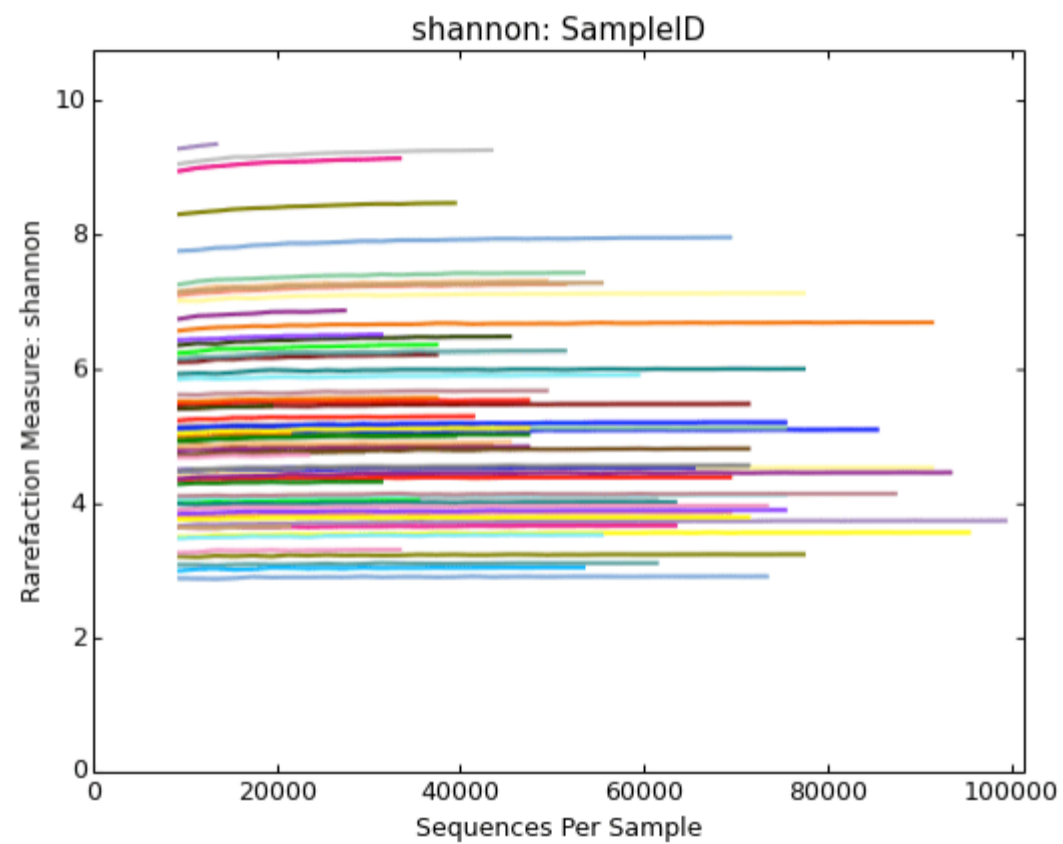**C**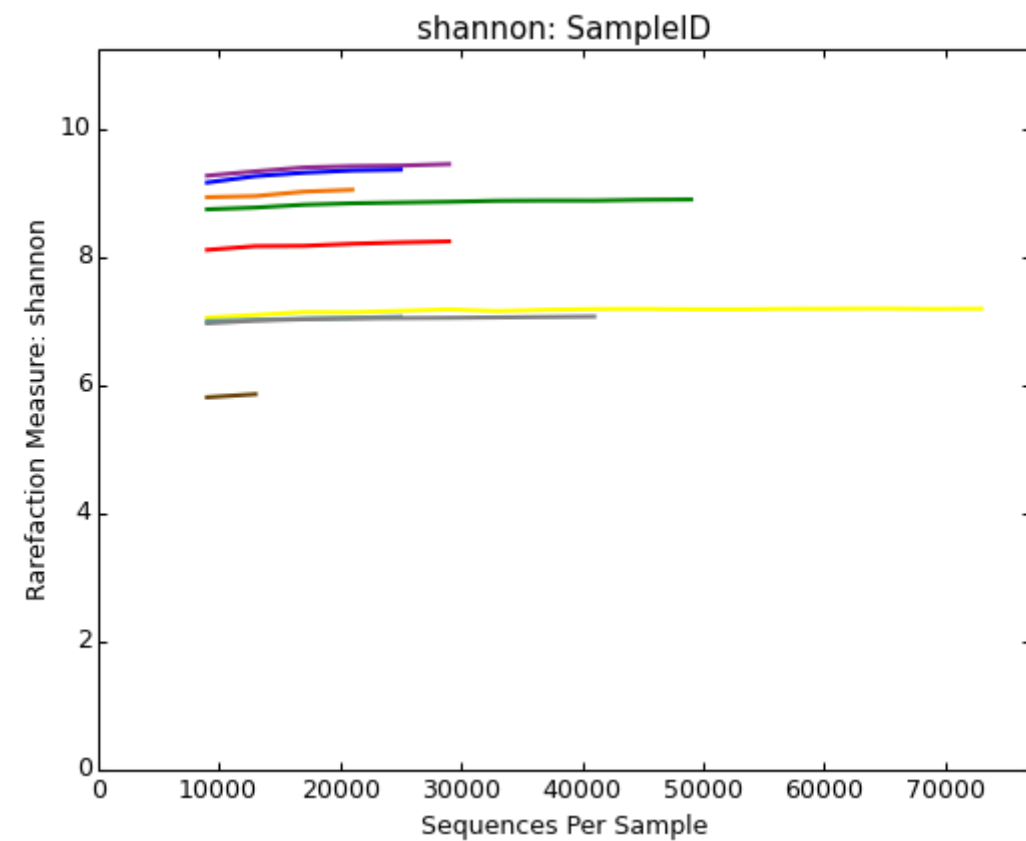**B**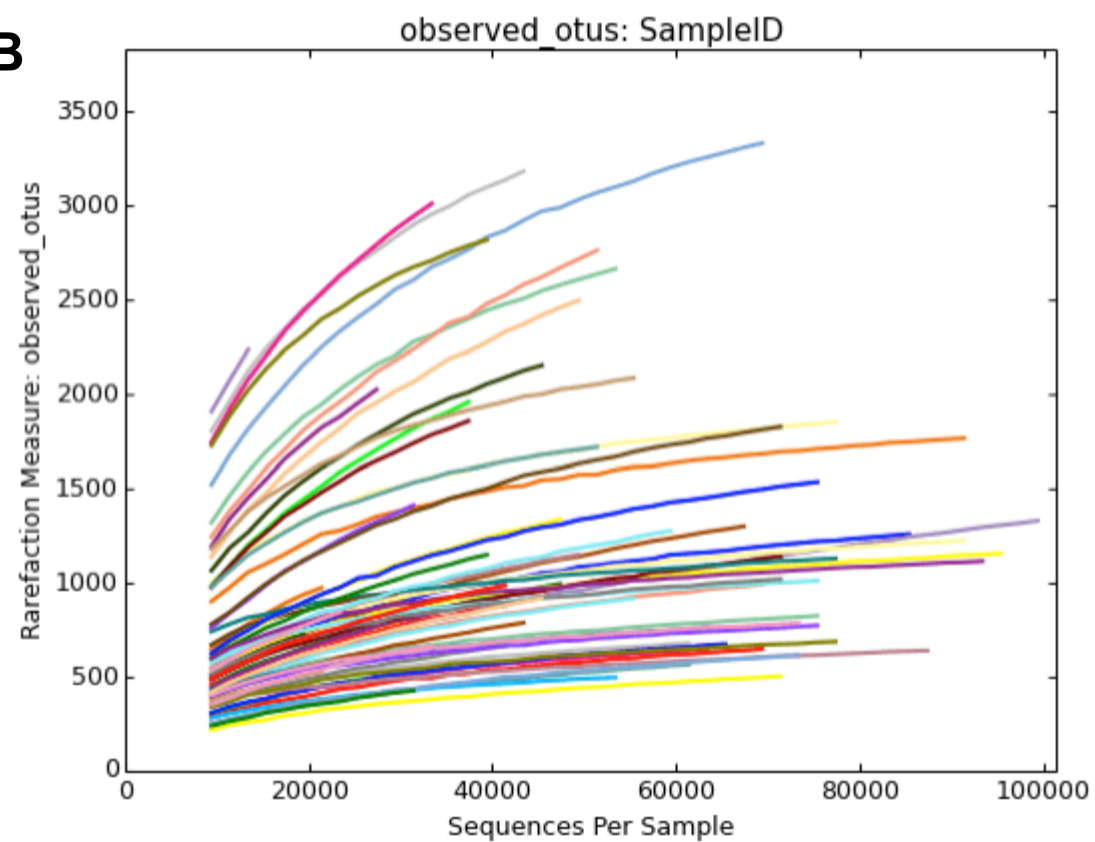**D**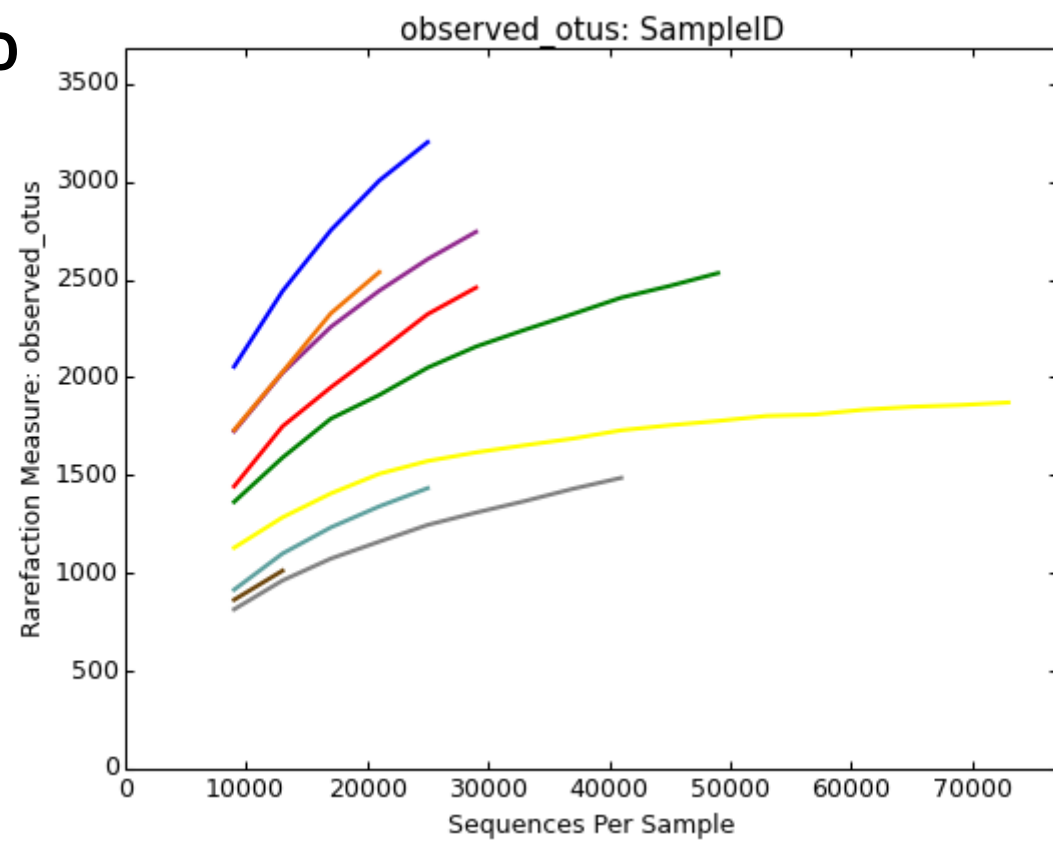

Supplement: Supplementary file 4 — Rarefaction curves of alpha diversity calculated on multiple rarefied data table for each of the 66 bat skin microbiota samples and 11 environmental samples. (A) Shannon diversity of bat skin samples. (B) Overall richness (OTUs observed) of bat skin samples. (C) Shannon diversity of environmental samples. (D) Overall richness (OTUs observed) of environmental samples. [file 40168_2017_334_MOESM4_ESM.pdf]

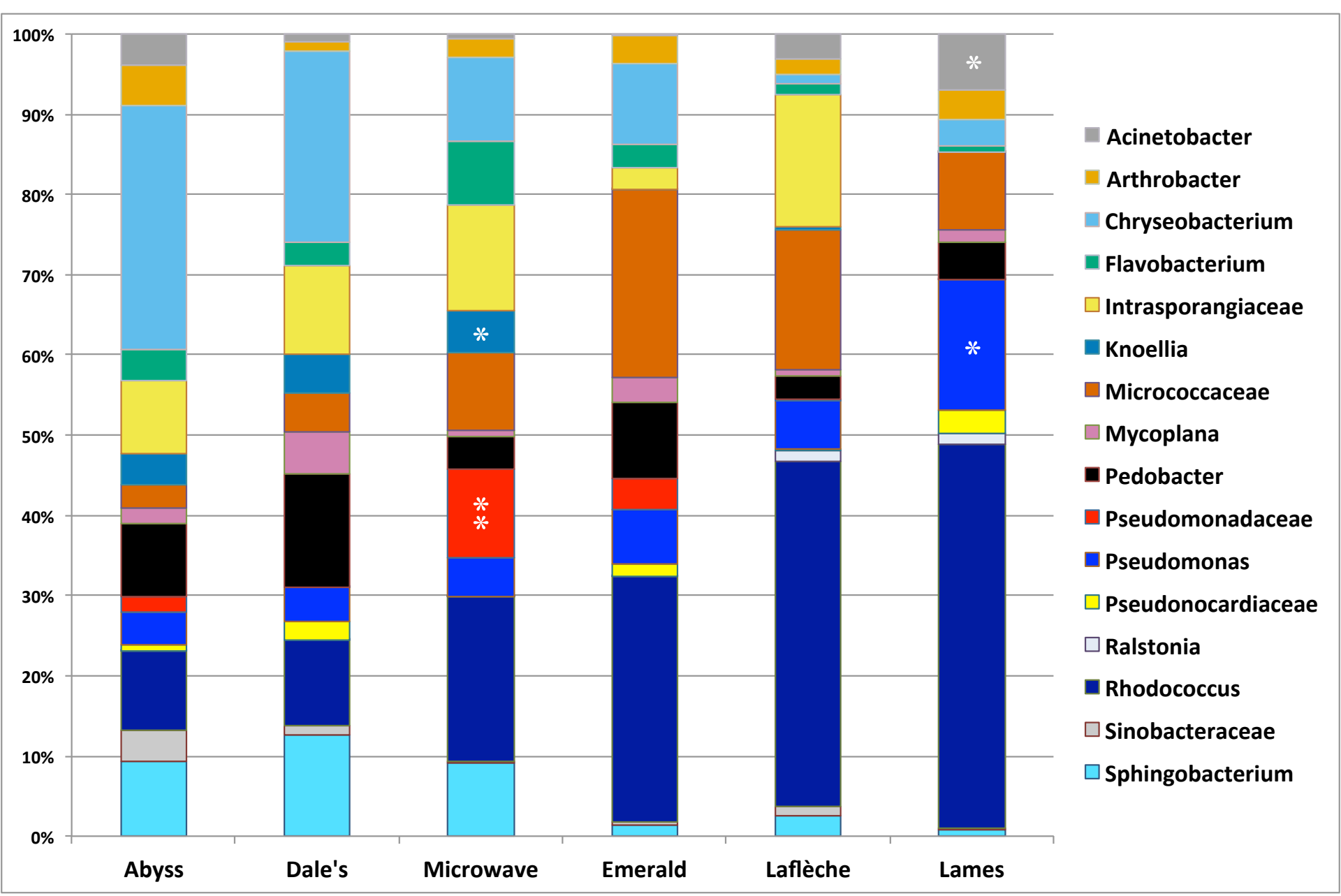

Supplement: Supplementary file 7 — Major bacterial taxa identified in bat skin microbiota samples. The 16 more abundant taxa across all hibernacula are provided. Stars represent significant indicator taxa. *IndVal < 0.50, **IndVal ≥ 0.50, ***IndVal ≥ 0.89. [file 40168_2017_334_MOESM7_ESM.pdf]
