# Supplementary material for: Enrichment of beneficial bacteria in the skin microbiota of bats persisting with white-nose syndrome
Source: Microbiome. 2017 Sep 5;5:115. doi: 10.1186/s40168-017-0334-y (PMC5584028; doi:10.1186/s40168-017-0334-y)
Supplement: Supplementary file 8 — M. lucifugus skin microbiota taxa indicator test and related association measure (A, B) of six hibernaculum groups with different WNS status in Canada. Indicator value tests were computed with the multipatt() function of the indicspecies package in R. Only taxa with A ≥ 0.4 were retained as indicators. A, the specificity, is the probability that a site belongs to the group given the fact that the species is found and B, the fidelity, is the probability of finding a given taxon when the sites belong to that group. *p ≤ 0.05, **p ≤ 0.01, ***p ≤ 0.001. [file 40168_2017_334_MOESM8_ESM.docx]

**Additional file 8:** *M. lucifugus* skin microbiota taxa indicator test and related association measure (*A, B*) of six hibernaculum groups with different WNS status in Canada. Indicator value tests were computed with the *multipatt*() function of the indicspecies package in R. Only taxa with *A* ≥ 0.4 were retained as indicators.

| **Hibernaculum group** | **Associated taxa** | ***A*** | ***B*** | ***IndVal*** | **p-value** | **Holm corrected p-value** |
| --- | --- | --- | --- | --- | --- | --- |
| Abyss | *Nitrosovibrio* | 0.892 | 1.000 | 0.945 | 0.0001 *** | 0.0026** |
|  | Cytophagaceae | 0.630 | 1.000 | 0.793 | 0.0011** | 0.0187* |
|  | Flavobacteriaceae | 0.425 | 1.000 | 0.652 | 0.0009 *** | 0.0162* |
| Microwave | Pseudomonadaceae | 0.635 | 1.000 | 0.797 | 0.0001 *** | 0.0026* |
|  | Brucellaceae:Other | 0.416 | 1.000 | 0.645 | 0.0024 ** | 0.0336* |
|  | *Microbacterium* | 0.413 | 1.000 | 0.643 | 0.0002 *** | 0.0044** |
|  | *Knoellia* | 0.408 | 1.000 | 0.639 | 0.0012 ** | 0.0192* |
| Lames | *Acinetobacter* | 0.450 | 1.000 | 0.671 | 0.0003 *** | 0.0060** |
|  | *Pseudomonas* | 0.392 | 1.000 | 0.626 | 0.0001 *** | 0.0026** |

*A*, the specificity, is the probability that a site belongs to the group given the fact that the species is found and *B*, the fidelity, is the probability of finding a given taxon when the sites belong to that group. ∗*p* ≤ 0.05, ∗∗*p* ≤ 0.01, ∗∗∗*p* ≤ 0.001.
