# Supplementary material for: Enrichment of beneficial bacteria in the skin microbiota of bats persisting with white-nose syndrome
Source: Microbiome. 2017 Sep 5;5:115. doi: 10.1186/s40168-017-0334-y (PMC5584028; doi:10.1186/s40168-017-0334-y)
Supplement: Supplementary file 9 — M. lucifugus skin microbiota taxa indicator and related association measure (A, B) of WNS-positive (Québec) and WNS-negative (Manitoba) sites in Canada. Indicator value tests were computed with the multipatt() function of the indicspecies package in R. Only taxa with A ≥ 0.4 were retained as indicators. A, the specificity, is the probability that a site belongs to the group given the fact that the species is found and B, the fidelity, is the probability of finding a given taxon when the sites belong to that group. *p ≤ 0.05, **p ≤ 0.01, ***p ≤ 0.001. [file 40168_2017_334_MOESM9_ESM.docx]

**Additional file 9:** *M. lucifugus* skin microbiota taxa indicator and related association measure (*A, B*) of WNS-positive (Québec) and WNS-negative (Manitoba) sites in Canada. Indicator value tests were computed with the *multipatt*() function of the indicspecies package in R. Only taxa with *A* ≥ 0.4 were retained as indicators.

| **WNS status and Province group** | **Associated taxa** | ***A*** | ***B*** | ***IndVal*** | **p-value** | **Holm corrected p-value** |
| --- | --- | --- | --- | --- | --- | --- |
| **WNS positive**  **sites**  **(Québec)** | *Ralstonia* | 0.997 | 0.879 | 0.936 | 0.0001 *** | 0.0026 ** |
|  | *Janthinobacterium* | 0.808 | 0.939 | 0.871 | 0.0023** | 0.0288 * |
|  | *Rhodococcus* | 0.751 | 1.000 | 0.867 | 0.0001 *** | 0.0026 ** |
|  | Micrococcaceae | 0.750 | 1.000 | 0.866 | 0.0001 *** | 0.0026 ** |
|  | *Pseudomonas* | 0.720 | 1.000 | 0.849 | 0.0001 *** | 0.0026 ** |
| **WNS negative**  **sites**  **(Manitoba)** | *Knoellia* | 0.979 | 1.000 | 0.989 | 0.0001 *** | 0.0026 ** |
|  | Brucellaceae:Other | 0.986 | 0.970 | 0.978 | 0.0001 *** | 0.0026 ** |
|  | *Nitrosovibrio* | 0.992 | 0.970 | 0.981 | 0.0001*** | 0.0026 ** |
|  | Flavobacteriaceae | 0.997 | 0.909 | 0.952 | 0.0001 *** | 0.0026 ** |
|  | Enterobacteriaceae | 0.912 | 1.000 | 0.955 | 0.0001 *** | 0.0026 ** |
|  | *Microbacterium* | 0.906 | 1.000 | 0.952 | 0.0001 *** | 0.0026 ** |
|  | *Sphingobacterium* | 0.839 | 1.000 | 0.916 | 0.0001 *** | 0.0026 ** |
|  | Cytophagaceae | 0.831 | 1.000 | 0.911 | 0.0007 *** | 0.0091 ** |
|  | *Chryseobacterium* | 0.798 | 1.000 | 0.893 | 0.0001 *** | 0.0026 ** |
|  | Xanthomonadaceae | 0.778 | 1.000 | 0.882 | 0.0001 *** | 0.0026 ** |

*A*, the specificity, is the probability that a site belongs to the group given the fact that the species is found and *B*, the fidelity, is the probability of finding a given taxon when the sites belong to that group. ∗*p* ≤ 0.05, ∗∗*p* ≤ 0.01, ∗∗∗*p* ≤ 0.001.
